# Importance of population-based studies in clinical practice

**DOI:** 10.4103/0301-4738.73681

**Published:** 2011-01

**Authors:** George Ronnie, Ramesh Sathyamangalam Ve, Lokapavani Velumuri, Rashima Asokan, Lingam Vijaya

**Affiliations:** Glaucoma Project, Vision Research Foundation, Sankara Nethralaya, Chennai, India

**Keywords:** Blindness, India, population-based study, primary open angle glaucoma, primary angle closure glaucoma, risk factors

## Abstract

In the last decade, there have been reports on the prevalence of glaucoma from the Vellore Eye Survey, Andhra Pradesh Eye Diseases Survey, Aravind Comprehensive Eye Survey, Chennai Glaucoma Study and West Bengal Glaucoma Study. Population-based studies provide important information regarding the prevalence and risk factors for glaucoma. They also highlight regional differences in the prevalence of various types of glaucoma. It is possible to gather important insights regarding the number of persons affected with glaucoma and the proportion with undiagnosed disease. We reviewed the different population-based studies from India and compare their findings. The lacunae in ophthalmic care that can be inferred from these studies are identified and possible reasons and solutions are discussed. We also discuss the clinical relevance of the various findings, and how it reflects on clinical practice in the country. Since India has a significantly high disease burden, we examine the possibility of population-based screening for disease in the Indian context.

Glaucoma is a major cause of blindness. It has been identified to be the second most common cause of blindness worldwide. In a recent publication, about 60 million persons are estimated to be affected by glaucoma.[1,2] Of these, an estimated 11.2 million cases are from the Indian subcontinent.[3]

There are regional differences in the prevalence of different types of glaucoma, and the way it presents.[3] From India, the prevalence and risk factors for glaucoma have been reported from several population-based studies. These include the Vellore Eye Survey (VES),[4] Andhra Pradesh Eye Diseases Survey (APEDS),[5,6] Aravind Comprehensive Eye Survey (ACES),[7] Chennai Glaucoma Study (CGS),[8–11] and West Bengal Glaucoma Study (WBGS).[12] We attempted to summarize some of the findings and risk factors they report. There are methodologic differences between the studies and diagnostic variations in the disease definition. The more recent studies have used the International Society of Geographical and Epidemiologic Ophthalmology (ISGEO) classification proposed by Foster *et al*.[13] for use in population-based studies. The study criteria are summarized in Table 1 and the ISGEO criteria in Table 2.

**Table 1:** Summary of the different population-based studies from India

Study	Study period	Population studied	Age group	Number examined (response rate %)	Diagnostic criteria for glaucoma		
					Elevated IOP	Optic disc changes	Visual field defects
VES	1994	Urban	30-60	972 (50.3)	Yes/No	Yes	Yes
APEDS	1996-2000	Urban	All ages	10273 (87.3)	No	Yes	Yes/No
ACES	1995-97 Rural	40+	5150 (93.0)	No	Yes	Yes/No
CGS*	2001-03	Rural	40+	3924 (81.75)	No	Yes	Yes/No
CGS	2002-04	Urban	40+	3850 (80.20)	No	Yes	Yes/No
WBGS*	1998-99	Rural	50+	1324 (83.1)	No	Yes	Yes/No

* The CGS and the WBGS used the ISGEO[13] criteria (with minor modifications) to diagnose disease. An IOP level that exceeds the 99.5th percentile for a normal population is used to diagnose disease only when the optic disc cannot be visualized and visual fields are not possible. APEDS: The Andhra Pradesh Eye Disease Study, CGS: The Chennai Glaucoma Study, WBGS: West Bengal Glaucoma Study, VES: Vellore Eye Study, ACES: The Aravind Comprehensive Eye Survey, RUR: Rural, URB: Urban, CI: Confidence Interval.

**Table 2:** International Society for Geographical and Epidemiological Glaucoma (ISGEO) criteria

	Visual acuity	IOP and treatment	Optic disc	Field defect
Category 1: Structural and functional evidence	-	-	CDR or CDR assymetry ≥ 97.5^th^ percentile for the normal population. Neural retinal rim width reduced to ≤0.1 CDR (Superior: 11-1 o’clock or inferior: 5-7 o’clock)	Defect consistent with glaucoma
Category 2: Advanced structural damage with unproved field defect	-	-	CDR or CDR assymetry ≥ 97.5^th^ percentile for the normal population. Neural retinal rim width reduced to ≤0.1 CDR (Superior: 11-1 o’clock or inferior: 5-7 o’clock)	Subjects who have not completed Visual fields
Category 3	<3/60	IOP>99.5^th^ percentile of normal population	Optic disc not seen	Field test not done
	<3/60	Evidence of glaucoma filtration surgery or using antiglaucoma medication		

**Classification of primary angle closure glaucoma**				
	Criteria			
Primary angle closure suspect (PACS)	Appositional closure contact between peripheral iris and posterior trabecular meshwork (pigmented TM not seen ≥ 180 or 270°)			
Primary angle closure (PAC)	PACS together with features indicating that TM obstruction by peripheral iris (peripheral anterior synechiae, elevated IOP, iris whorling, glaucomflecken, lens opacities or extensive TM pigmentation			
Primary angle closure glaucoma (PACG)	PAC together with evidence of glaucoma (as defined above)			

PACS: Primary angle closure suspect, PACG: Primary angle closure glaucoma, PAC: Primary angle closure

## Primary Open Angle Glaucoma

**Figure 1:**
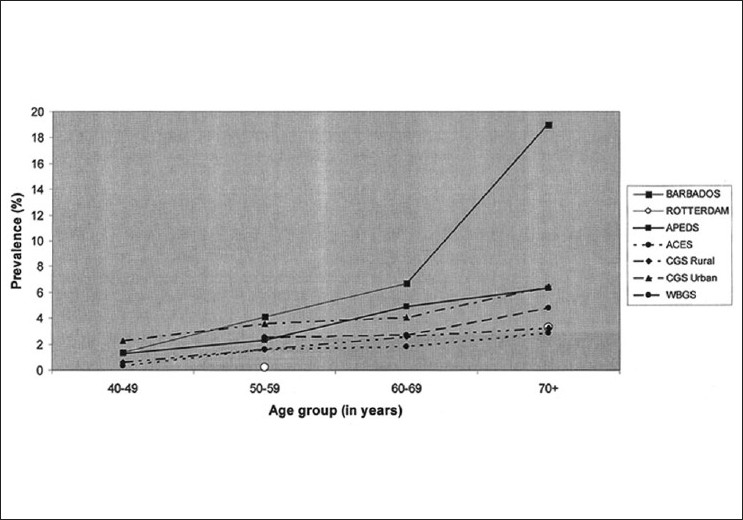
Comparison of POAG prevalence vs. age among populationbased studies. There is an increase in the prevalence of POAG reported in all the studies. The Barbados study showed large increase in the prevalence of POAG over 60 years of age. The Rotterdam study reported age wise prevalence for only two groups. (APEDS: The Andhra Pradesh Eye Disease Study, CGS: The Chennai Glaucoma Study, WBGS: West Bengal Glaucoma Study, VES: Vellore Eye Study, ACES: The Aravind Comprehensive Eye Survey, RUR: Rural, URB: Urban, CI: Confidence Interval, POAG: Primary open angle glaucoma)

The reported prevalence for Primary Open Angle Glaucoma (POAG) varies between 1.62% and 3.51%[4,6–8,10,12] [Table 3]. A trend toward higher prevalence was noted in the urban cohorts studied. Among the risk factors reported, increasing age was a consistent risk factor for POAG across all studies[4,6,7,8,10,12] [Fig. 1]. The Fig. 1 also compares the POAG prevalence with increasing age between all the population based from India and two international studies (The Barbados study[14] and the Rotterdam study[15]).

**Table 3:** Prevalence of Glaucoma in different studies

	APEDS (*n* = 934)	ACES (*n* = 5150)	CGS Rural (*n* = 3924)	CGS Urban (*n* = 3850)	WBGS (*n* = 1269)
POAG	2.56 (1.22, 3.92)	1.7 (1.3, 2.1)	1.62 (1.42, 1.82)	3.51 (3.04, 4.0)	2.99
PACG	1.08 (0.36, 1.80)	0.5 (0.3, 0.7)	0.87 (0.58, 1.16)	0.88 (0.6, 1.16)	0.24
PAC	NR	NR	0.71 (0.45, 0.98)	2.75 (2.01, 3.49)	NR
PACS	2.21 (1.15, 3.27)	NR	6.27 (5.51, 7.03)	7.24 (6.58, 8.02)	NR

VES reported POAG prevalence (30-60 years): 0.41% (95% CI: 0.08, 0.81) and PACG prevalence (30-60 years): 4.32 (95% CI: 3.01, 5.63), ACES reported PACG prevalence (40 years or more): 0.5% (95% CI: 0.3, 0.7). APEDS: The Andhra Pradesh Eye Disease Study, CGS: The Chennai Glaucoma Study, WBGS: West Bengal Glaucoma Study, VES: Vellore Eye Study, ACES: The Aravind Comprehensive Eye Survey, RUR: Rural, URB: Urban, CI: Confidence Interval. POAG: Primary open angle glaucoma, PACG: Primary angle closure glaucoma, PAC: Primary angle closure and PACS: Primary angle closure suspect

### Risk factors for POAG

1. *Race*: Forty-six population-based studies were analyzed in a meta-analysis by Rudnicka *et al*.[16] to evaluate the effect of race on POAG prevalence. The pooled prevalence estimate for POAG was 1.4% (95% CI: 1.0-2.0%) in Asian populations, 4.2% in Black populations (95% CI: 3.1-5.8%), and 2.1% (95% CI: 1.6-2.7%) in White populations.
2. *Age*: As with most chronic diseases, the prevalence of disease increases with increasing age due to the increase in the cumulative number of persons with disease. This increase appears to be exponential in western populations as compared to Asian reports.[1,14,17] In the CGS (Urban),[8,10] the risk of disease in those above the age of 70 years was five times that of the 40-49 age group. [Fig. 1]
3. *Intraocular pressure (IOP)*: Elevated IOP is no longer considered to be a diagnostic criterion for POAG. Most population-based studies report that between 30% and 60% of the subjects diagnosed to have POAG actually have an IOP recording in the statistically normal range.[3–12] On further visits, the IOP may be recorded to be greater than normal. This highlights the danger of considering a “normal” IOP in isolation while assessing the risk of POAG in an individual. While many of those with POAG have normal IOP, the risk of having POAG increases dramatically with increase in IOP. This is because among those with elevated IOP a large proportion will have POAG (the rest being ocular hypertensive or pre-perimetric disease), while those with POAG and a normal presenting IOP will form only a small proportion of all those with normal IOP even if they are a substantial percentage of those with the disease.
4. *Myopia*: Myopia has been an inconsistent risk factor for POAG as identified by only few studies.[16]
5. *Central corneal thickness (CCT)*: Those with POAG in the Rotterdam Study were reported to have thinner CCT than normal.[15] The Barbados Eye Studies[14] participants with POAG had thinner corneas (520.6 ± 37.7 μm) than those classified as non-glaucomatous (530.0 ± 37.7 μm). From the CGS, the only study from India that reported CCT data, the mean CCT in POAG subjects was not significantly different from that of the normal study population.[17]
6. *Diabetes mellitus*: The Blue Mountains[18] and Beaver Dam[19] studies reported that diabetes mellitus was a risk factor for POAG. However, the Baltimore Eye survey[20] did not find any relationship.
7. *Hypertension*: The relationship between systemic hypertension and POAG has been reported in few studies (Baltimore[20] and Rotterdam[15]) with hypertensives being at greater risk for POAG. However, this is again not consistently seen in all reports.[4,6–8,10,12]
8. *Family history*: It is difficult to assess the true association with family history and POAG in population-based studies. Since only 10-60% of those with glaucoma have been diagnosed; a family history of no glaucoma may be inaccurate.[21] When all first degree relatives of those diagnosed to have POAG from the Rotterdam study were examined, 22.4% of them were found to have POAG.[15] This is nearly 10 times greater than the risk in the general population.

## Primary Angle Closure Disease = Primary Angle Closure Suspects + Primary Angle Closure + Primary Angle Closure Glaucoma

The clinical suspicion that angle closure glaucoma was a significant cause of ocular morbidity in the country was confirmed by the VES, which was the first population-based glaucoma prevalence study from India.[4] Jacob *et al*.[4] reported that 10.3% of the population had occludable angles or angle closure glaucoma. Subsequent studies reported a substantial proportion of angle closure glaucoma. However, there were wide variations in the reported rates for PACS and PAC[3–5,7,9,11,12] [Table 3]. These differences could be related to diagnostic criteria as well as gonioscopic technique and the use of standard testing conditions (dim illumination, a shortened slit beam that does not fall on the pupil). Studies that were carried out by persons with specialized glaucoma training have consistently reported higher rates of PACS and PAC.[4,8,9]

There were diagnostic differences between the studies. For angle closure disease, both the VES and the CGS used non-visibility of the filtering trabecular meshwork for 180° or more of the angle to define angle closure. The VES[4] classified those with PAC and PACG as PACG, the APEDS[5] classified those with IOP greater than 22 mmHg or IOP, disc and field changes in those with occludable angles (270° or more of the angle narrow) as having PACG. On the basis of ISGEO[13] definitions, this would include some of those now classified as PAC but would exclude those with synechial closure in the absence of raised IOP, disc or field changes, potentially resulting in underestimation of the prevalence of PAC and PACG combined. The ACES[7] defined PACG if it met at least two of the criteria of glaucomatous optic disc damage or glaucomatous visual field defects in combination with anterior chamber angle partly (9’o clock hours) or totally closed, appositional angle closure or synechiae in the angle along with the absence of signs of secondary angle closure.

### Risk factors for PACG

**Figure 2:**
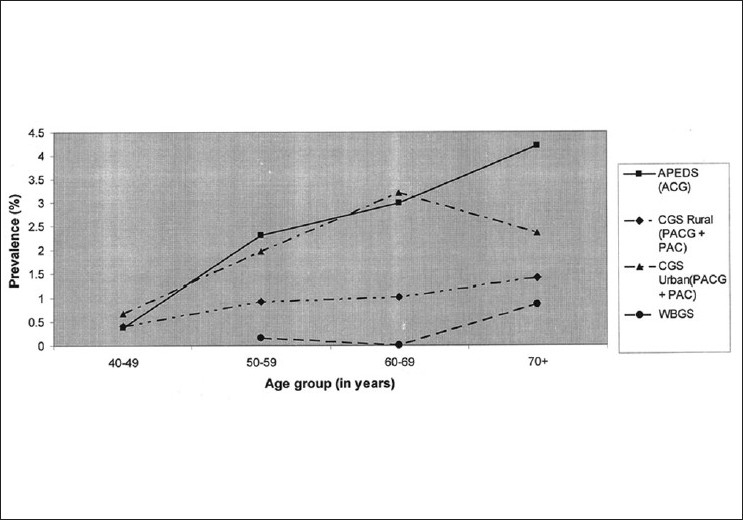
Comparison of PACG prevalence vs. age among populationbased studies from India. (APEDS: The Andhra Pradesh Eye Disease Study, CGS: The Chennai Glaucoma Study, WBGS: West Bengal Glaucoma Study, VES: Vellore Eye Study, ACES: The Aravind Comprehensive Eye Survey. RUR: Rural, URB: Urban, CI: Confidence Interval, PACG: Primary angle closure glaucoma)

1. *Symptoms*: Most angle closure disease in India are asymptomatic.[3–5,7,9,11,12] The vast majority of patients have the chronic form of disease which does not present with significant visual symptoms. The presentation may be different from that of persons of Chinese origin who are more likely to present with acute symptoms.[22,23]
2. *Ethnicity*: The prevalence of angle closure glaucoma shows much wider variations than for open angle glaucoma. The highest rates have been reported among Eskimos.[24] High prevalence has also been reported from China, Mongolia, Southeast Asia, and India.[3–5,7,9,11,12,22,25–28]
3. *Age*: Increasing age [Fig. 2] is a risk factor for PACG too. However, the increase does not appear to follow the exponential curve described for POAG. An exponential increase in prevalence was noted in the CGS (Urban cohort) for PACS with increasing age. Lenticular changes could be responsible for this finding.[3,29]
4. *Biometry*: Eyes with angle closure disease appear to have a shorter axial length, a shallower anterior chamber, and a thicker lens than the normal population.[5,29,30] All these factors contribute to a crowded anterior segment of the eye. In this situation, small increases in lens thickness or decrease in the anterior chamber depth would result in greater iris convexity and consequently a narrower angle recess.
5. *Gender*: Female gender has been reported to be an independent risk factor for angle closure glaucoma and for angle closure disease. This is possibly related to biometric differences between genders since women appear to have shorter eyes and a shallower anterior chamber depth than men.[29,30]
6. *Hyperopia*: An association of increasing hyperopia with the angle closure disease would be expected taking into account that hyperopic eyes are likely to be shorter and therefore at greater risk of angle closure disease. This has not been reported consistently from population-based studies.[3–5,7,9,11,12,23,25–28] One possible explanation is related to the cataractous changes occurring in these eyes. Minor degrees of nuclear sclerosis are known to induce a myopic shift in refraction values. Since nuclear sclerosis is common among the study population and among those with angle closure disease, this myopic shift could confound any possible association with hyperopia.

Some of the studies have reported the prevalence of secondary glaucoma. The WBGS[12] reported a rate of 0.08%, APEDS[6] 0.21% (among those aged 30 and above), and 0.3% in the ACES, respectively.[7] Absolute glaucoma was diagnosed in 0.06% of those examined in the ACES.[31] The prevalence of pseudoexfoliation (PXF) has also been reported by the rural arm of CGS[32] and the APEDS,[33] the reported prevalence of PXF was 3.8% and 3.01% and PXF glaucoma was 13% and 5.5%, respectively. In the rural arm of the CGS, 1.38% of the population had glaucoma with aphakia or pseudophakia.[34] An additional 0.49% had PXF glaucoma.[32] The ACES reported that the prevalence of PXF glaucoma was 0.44%.

## Blindness Due to Glaucoma

Glaucoma is the second leading cause of blindness in the adult population in India.[35] Angle closure glaucoma causes blindness in a greater proportion of affected individuals than that of open angle glaucoma. The rates of bilaterally blind because of POAG in the APEDS,[6] ACES,[7] CGS (rural),[8] CGS (Urban),[10] and WBGS[12] were 11.1%, 1.6%, 3.2%, 1.5%, and 5.2%, respectively. For angle closure glaucoma, the blindness rates for APEDS,[5] CGS (rural),[9] and CGS (Urban)[11] were 16.6%, 2.9%, and 5.9%, respectively. The WBGS[12] reported only three cases of PACG and none of whom were blind. PACG caused two times the proportion of bilateral blindness than that of POAG.[36]

## Incidence of Glaucoma

Thomas *et al*. reported the 5-year incidence figures from the VES for angle closure,[37] angle closure glaucoma,[38] and POAG.[39] Among ocular hypertensive 17.4% (95% CI: 1.95-32.75) progressed to POAG (3.5% per year). Among the 110 normals, one progressed to normal tension glaucoma (NTG). The relative risk of progression among ocular hypertension (OHT) was 19.1 (95% CI: 2.2-163.4). All those who progressed had bilateral OHT. Bilateral OHT, higher peak IOP, and large diurnal variation were noted to be risk factors for progression.[39]

For angle closure disease 22% (95% CI: 9.8-34.2) of PACS re-examined after a 5-year period progressed to PAC (seven synechial and four appositional); none of them developed disc or visual field changes.[37]

The relative risk for progression among PACS was 24 (95% CI: 3.2-182.4). There was no significant difference in axial length, anterior chamber depth, or lens thickness between those who progressed and those who did not. Bilateral PACS was a clinical risk factor for progression. Primary angle closure was noted to progress to PACG in 28.5% (95% CI: 12-45%).[38] Progression to PACG was based on the optic disc damage and visual field defects on automated perimetry. One-third of cases had undergone laser peripheral iridotomy at baseline, the others had refused laser. At the 5-year follow-up, 11% of those who underwent iridotomy progressed as compared to 36.8% of those who did not.[38] There was no significant difference in biometric parameters between the progressed and non-progressed groups.

## Disease Estimates from Population-Based Studies

Another estimate of the number of persons with glaucoma and at risk of disease in the country was recently reported.[3] An estimated 6.48 (95% CI: 5.06-7.89) million person were estimated to have POAG, estimates for OHT were 4.7 (95% CI: 3.94-9.31) million. PACG affects an estimated 2.54 (95% CI: 1.88-4.28) million persons. Those with some evidence of damage to the trabecular meshwork such as raised IOP or peripheral anterior synechiae (PAS) or glaucomatous optic disc or visual field changes comprise 6.62 (95% CI: 4.78-9.41) million persons. The estimated total number of persons with angle closure disease is 27.66 (95% CI: 24.00-30.92) million. The prevalence of PACG and PAC show a linear increase with age [Fig. 2]. The secondary glaucomas could affect 2.28 million persons.[3] These figures are disturbing considering that most of the glaucoma in the country is undiagnosed and that the majority of disease is undetected.[3,40]

In all population-based studies, the majority of those diagnosed in the study with glaucoma were previously undetected, previous diagnosis rates range from 6% to 17%.[3–12] These rates are far below the 40-60% of previously diagnosed glaucoma from western studies.[14–16,18–20] These poor detection rates are a cause for concern, since with greater life expectancy resulting in an aging population the number of those affected with glaucoma is expected to increase to 60 million by 2010.[2] The poor detection rates are even a greater cause for concern when we look at data reported from the ACES and CGS. The ACES[7] reported that even though 50% of those diagnosed to have POAG in their study cohort had undergone an eye examination by an ophthalmologist in the past, <20% of them had been detected to have disease prior to the study evaluation. While none of them were examined in the year prior to the study examination, it is likely that they would have had some detectable signs of glaucoma at the time of the earlier examination. From the CGS reported prevalence of glaucoma in the urban population, of the five persons who had been diagnosed and treated for glaucoma, two (40%) actually had angle closure glaucoma.[10] It appears from these findings that we are not detecting glaucoma in our clinics and, if we detect it, we are misdiagnosing angle closure glaucoma. It is possible that this is related to the overemphasis on elevated IOP being essential for glaucoma diagnosis. The use of elevated IOP to diagnose glaucoma carries even greater risks when IOP is not measured using applanation tonometry. In many settings, Schiotz is still used to measure IOP. The use of a non ideal technique itself will further reduce the diagnostic capability of IOP measurement alone.[41,42] Unless optic disc evaluation is incorporated into the clinical routine, we would continue to have poor detection rates. The misdiagnosis of angle closure glaucoma points to either poor gonioscopy technique or not performing the test. In a country which has a substantial number with angle closure disease this is unacceptable.

Poor examination techniques could also be related to poor primary training. In a recent article, Thomas *et al*.[43] reported major lacunae in the residency training programs that they evaluated. These centers had been upgraded with equipment and provided training of trainers. In spite of these measures, ophthalmology training imparted was substandard. Since the majority of those with glaucoma would present to the general ophthalmologist, it is imperative that we take steps to improve basic training. Providing a sound foundation at the time of basic training would enhance quality of eye care across the country. While there are excellent glaucoma fellowship training programs available across the country, the number of ophthalmologists with fellowship training is small and they are not likely to contribute significantly to disease detection.

Other eye care personnel in the country (ophthalmic assistants and optometrists) contribute little to detection of disease in the country.[44] With few exceptions, most optical dispensaries offer no additional examination beyond refraction and spectacle prescription. There are even greater disparities in training for an optometry degree or diploma; the duration of the course ranges from 6 months to 4 years with larger variations in the quality of training. The optometrist is a primary contact for many of those who require spectacles and enhancing quality of examination at this level would add a valuable primary care point.[44]

Reports from population-based studies highlight very poor awareness about glaucoma in the population.[40,45,46] Awareness about glaucoma ranged from 0.27% in the rural population of the ACES study[46] to 13.3% in the urban cohort of the CGS.[40] These are much lower than the rates reported from Australia (70-92%)[40] and United States of America (72-81%).[40] This may partly contribute to the poor detection rates for glaucoma. Increasing public awareness should automatically result in greater number of persons seeking eye evaluation. However, unless detection rates in our clinics are improved this need not translate to a reduction of the numbers with undetected disease. Access to eye care is another obstacle that will have to be overcome in order to improve diagnosis rates.[47] Robin *et al*.[48] report from the ACES that even though three-fourths of persons aged 40 years or older in the rural population required eye care services only one-third had undergone an eye examination at any time in their lives.

**Figure 3:**
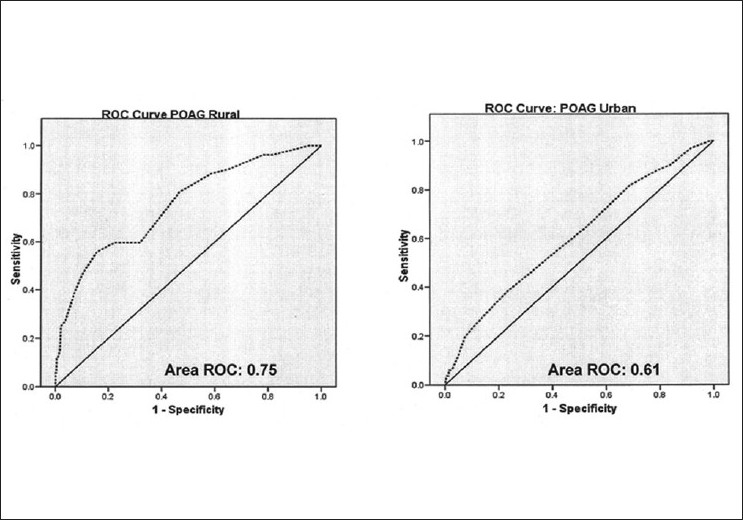
Area under ROC for IOP among CGS:POAG subjects. Diagnostic ability of intraocular pressure measurements among POAG subjects. (CGS: The Chennai Glaucoma Study, POAG: Primary open angle glaucoma, AROC: Area under receiver operated characteristic curve)

In the presence of the combination of a large population with glaucoma and low diagnosis rates in the country, screening is often considered to be an attractive option. While glaucoma meets many of the criteria for a disease for which screening could be considered such as the significant prevalence and a pre-symptomatic phase, there is a lack of an accepted screening test [Table 4] for the disease.[49,50] IOP on its own has very poor sensitivity and specificity [Fig. 3] to diagnose POAG.[51] The frequency doubling perimetry (FDP) in the screening mode shows promise.[52] However, it is not specific for glaucoma and visual field defects could be because of other neuro-ophthalmological or retinal causes. The optic nerve head and nerve fiber layer imaging devices do not perform much better in detecting glaucoma.[53,54] The devices have sensitivity and specificity values in the mid eighties which are inadequate for population-based glaucoma detection.[53,54] Screening methods to detect PACD have similar limitations.[55–57] Among the non contact screening methods, van Herick’s grading has reasonable sensitivity and specificity to screen for angle closure disease,[56] establishment of the diagnosis in the clinic will require gonioscopy performed under standard conditions (using a shortened slit beam that does not fall on the pupil) and of course IOP measurement and optic disc evaluation. The non-contact technique [Table 5] such as scanning peripheral anterior chamber depth analyzer (SPAC) and anterior segment optical coherence tomography (AS OCT) have poor specificity and are inappropriate for screening because of the high false positive rates.[58–60]

**Table 4:** Sensitivity and specificity of diagnostic tests for POAG

Test	Sensitivity	Specificity
Tonometry (at cutoff IOP > 21 mmHg)[49,51]	25.1-47.1%	92.4-95.3%
Optic disc examination (CDR ≥ 0.55)[53]	59%	73%
Automated perimetry[50]	97%	84%
Frequency doubling technology[52]	90-94%	91-96%
Sterephotographs[53]	94	87
HRT II[54]	73, 84	77, 90
OCT 3, RNFL[54]	86,82	84,84
GDx VCC[54]	84	84

POAG: Primary open angle glaucoma, HRT: Heidelberg Retinal tomography, OCT: Optical coherence tomography, RNFL: Retinal nerve fiber layer analysis, GDx VCC: Scanning laser polarimetry

**Table 5:** Sensitivity and specificity of diagnostic tests for PACD

Test	Sensitivity (%)	Specificity (%)
Oblique flashlight test[55,56]	80-86	69-70
VH grade ≤ 2[57,58]	62-80	89.3-92
AS-OCT[59]	94.1	55.3
SPAC[60]	84.9	73.1

VH: van Herick grading, AS-OCT: Anterior segment Optical coherence tomography, SPAC: Scanning Peripheral Anterior Chamber Depth Analyzer, PACD: Primary Angle Closure Disease

With high false positive rates expected for most tests due to the 5% disease rate in the population use of these tests will result in large numbers of false positives. They will have to be evaluated in detail at a tertiary care center and may need to travel long distances for confirmatory tests. The majority of the test positives will be false positives, this is likely to adversely impact any screening program since most of those referred for further evaluation would turn out to be normal. This would adversely affect future participation in the program. The problems of “labeling” persons who may not have access to care, resulting in needless anxiety, also needs to be taken into consideration.

## Conclusions

The population-based studies report very poor diagnosis rates for the disease and also highlight the poor diagnostic value of a single IOP recording. The necessity of actively looking to diagnose the disease becomes imperative when we consider that one in eight persons above the age of 40 years in India is either suffering from glaucoma or is at risk of the disease. The lack of any simple screening techniques only reinforces the fact that performing a comprehensive eye evaluation on every person who enters the eye care system is the only method of consistently detecting glaucoma. It is apparent that current standards of evaluation are suboptimal. Improving awareness about the disease and the required standards for examination among the general public may be the most effective way of ensuring that such evaluations become a part of the routine patient assessment in ophthalmic practice in the country.
